# Serum dioxin and DNA methylation in the sperm of operation ranch hand veterans exposed to Agent Orange

**DOI:** 10.1186/s12940-019-0533-z

**Published:** 2019-10-29

**Authors:** Karl T. Kelsey, Matthew Rytel, Edward Dere, Rondi Butler, Melissa Eliot, Susan M. Huse, E. Andres Houseman, Devin C. Koestler, Kim Boekelheide

**Affiliations:** 1 Department of Epidemiology, Brown University School of Public Health, Providence, RI 02912 USA; 2 Department of Pathology and Laboratory Medicine, Brown University School of Public Health, Providence, RI 02912 USA; 3 NIAID Collaborative Bioinformatics Resource, Frederick National Laboratory for Cancer Research, Frederick, MD 21701 USA; 4 La Center, USA; 5 Department of Biostatistics & Data Science, University of Kansas Medical Center, Kansas City, KS 66160 USA

**Keywords:** Sperm, Dioxin, Agent Orange, DNA methylation, Epigenetics

## Abstract

**Background:**

Exposure to the herbicide Agent Orange during the Vietnam War was widespread and is associated with numerous adverse health outcomes. A continuing concern of veterans is the possibility that exposure to the dioxin-containing herbicide might induce adverse reproductive outcomes. We sought to assess whether exposure to Agent Orange in Vietnam was associated with changes in DNA methylation in sperm in a subset of Vietnam veterans who participated in the Air Force Health Study (AFHS).

**Methods:**

We studied 37 members of the AFHS chosen to have no, low, medium or high exposure to Agent Orange, based upon serum dioxin levels obtained during a series of examinations. DNA from stored semen was extracted and DNA methylation assessed on the Illumina 450 K platform.

**Results:**

Initial epigenome-wide analysis returned no loci that survived control for false discovery. However, the *TEAD3* gene had four different CpG sites that showed loss of DNA methylation associated with dioxin exposure. Analysis assessing regional DNA methylation changes revealed 36 gene regions, including the region of the imprinted gene *H19* to have altered DNA methylation associated with high exposure compared to the low exposure group. Additional comparison of our data with sperm DNA methylation data from Russian boys exposed to dioxin found an additional 5 loci that were altered in both studies and exhibited a consistent direction of association.

**Conclusions:**

Studying a small number of sperm samples from veterans enrolled in the AFHS, we did not find evidence of significant epigenome-wide alterations associated with exposure to Agent Orange. However, additional analysis showed that the *H19* gene region is altered in the sperm of Agent Orange-exposed Ranch Hand veterans. Our study also replicated several findings of a prior study of dioxin-exposed Russian boys. These results provide additional candidate loci for further investigation and may have implications for the reproductive health of dioxin-exposed individuals.

## Introduction

Agent Orange was an herbicide/defoliant that was sprayed across Vietnam and Southeast Asia by the U.S. Air Force during the war in Vietnam as part of a mission termed Operation Ranch Hand. The intent of this widespread use was to clear vegetative cover used by the enemy as well as to destroy crops that fed them. The most toxic form of dioxin – 2,3,7,8-tetrachlorodibenzo-*p*-dioxin (TCDD) – was present in Agent Orange (AO) as a byproduct of the industrial processes employed to manufacture the herbicide. The soldiers who were tasked with receiving, storing, distributing, mixing and applying Agent Orange (Ranch Hands) were heavily exposed to it, and thus at risk for health issues associated with exposure to TCDD. Dioxins in general, and TCDD in particular, are extremely lipophilic and thus persistent in both the individual and the environment. The half-life of TCDD in the human body has been estimated to range from 7 to 11 years [1, 2].

The health effects of exposure to AO have been assessed and reviewed by the National Academy of Sciences on an ongoing basis since 1994 [1]. The range of adverse health effects that have been associated with AO exposure is broad, including likely associations with soft-tissue sarcoma, non-Hodgkin lymphoma, chronic lymphocytic leukemia, Hodgkin lymphoma, chloracne, hypertension and monoclonal gammopathy of undetermined significance [2]. Controversy exists around the nature of any association between exposure to AO and subsequent F1 birth defects, but the most recent review concludes that “evidence remains inadequate or insufficient to determine whether there is an association between exposure to [agent orange] and birth defects in the children of Vietnam veterans” [2]. While the efforts to assess possible health effects have been intense, it is apparent that little data has been available to address the significant emerging concerns of Vietnam veterans around the possible health effects of paternal AO exposures on their descendants. To our knowledge, there are no data on AO exposure and subsequent reproductive outcomes or transgenerational health effects in Vietnam veterans specifically, and as the Veterans Agent Orange Committee notes in the most recent report “more work in this area is warranted”; it recommends “further specific study of the health of offspring of male Vietnam veterans” [2].

TCDD is a well-known endocrine disrupting chemical [3] that has recently been shown to alter the epigenome [4]. Considerable in-vitro evidence has demonstrated that TCDD is particularly epigenetically active in developing organisms [5–9] as well as in male germ cells (reviewed in [10]). Further, recent studies report that dioxin exposure (as well as other endocrine disrupting agents) can induce inter- and transgenerational alterations in sperm DNA methylation [11–14]. Exposure to TCDD also has been associated with poor semen parameters in exposed peripubertal boys [15]. Similar findings have been reported after childhood exposures to high levels of TCDD [16]. Recently, studying a cohort of Russian boys exposed to TCDD, Pilsner et al. [17] reported 666 CpGs to be aberrantly methylated in the sperm epigenome in the groups with the highest compared to the lowest serum TCDD concentrations.

Given the growing literature showing the potential for TCDD exposure to alter the germline epigenome, we have studied samples from veterans involved in Operation Ranch Hand. Intense interest in the possible health effects of exposure to AO in Vietnam prompted the Air Force, in 1982, to launch a study of the soldiers tasked with handling herbicides and defoliants in Vietnam, called the Air Force Health Study (AFHS), with an aim to determine the health effects these soldiers might experience as a consequence of their exposure. In 1996 Henriksen et al [18] reported on the reproductive health of these veterans and observed no testicular abnormalities, no alterations in sperm count or percentage of abnormal sperm in the AFHS associated with serum dioxin levels. However, as these measures are known to be poorly predictive of adverse reproductive outcomes, we focused upon the possible impacts of TCDD on the sperm epigenome, targeting our investigation on the induction of epigenetic changes in a subset of the Ranch Hand veterans. Additionally, with increasing evidence in the literature of dioxin-induced transgenerational alterations to the sperm epigenome, greater knowledge of the epigenetic changes that occurred in veterans exposed to Agent Orange is essential to understanding the potential impacts on their descendants.

## Methods

The Air Force Health Study is a prospective study that began in 1982, in which consenting veterans, over numerous visits (study ‘cycles’) received physical examinations, health questionnaires, medical record reviews, and biospecimen sampling [19]. The study included examinations that occurred in cycles at time points of 1, 3, 5, 10, 15, and 20 years after the initiation of the study [19]. Semen was collected from the entire AFHS cohort in 1982, and subsequently, serum dioxin assays were performed by the Centers for Disease Control on 777 Ranch Hands and 1174 control subjects during cycles 3 to 6 (1987–2002) of the study [20]. We received semen samples from cycles 2, 5 and 6, and isolated genomic DNA. The process of isolation of the semen has been described by the Vietnam Experience Study [21]. While somatic cells are not known to have been assessed in these samples, contamination will not be in excess of approximately 1% [22] and thus this is unlikely to affect the data. Genome-wide DNA methylation levels were assessed from the control group (non-Ranch Hand) and Ranch Hand personnel using the Illumina 450 K Infinium Methylation Beadchips. The raw data from the beadchips was processed and normalized, and met all quality control requirements. De-identified personnel information was used to determine potential cofounders, including age, smoking history, and body mass index (BMI). Exposure categories – defined according to lipid-adjusted serum levels of dioxin – were characterized as control (non-Ranch Hand control group with dioxin adjusted serum levels ranging from 2.21 to 7.05 ppt; *n* = 12), low (dioxin adjusted serum levels 5.6 to 8.0 ppt; *n* = 4), moderate (dioxin adjusted serum levels between 8.9 to 19.6 ppt; *n* = 11); and high (dioxin adjusted serum levels 26.6 to 167.6 ppt; *n* = 15). There were no participants with non-detectable dioxin levels. Subjects in the control group were stationed in Southeast Asia during the Vietnam War, and therefore were not exposed to Agent Orange [23].

Using the Infinium 450 K platform, we obtained DNA methylation measurements for each specimen; on this platform, DNA methylation is typically measured on an “average beta” scale, which represents the fraction of methylated molecules (for each target site) and is bounded by 0 (unmethylated) and 1 (methylated). We performed standard normalization and QA/QC procedures, including the removal of poor quality CpGs and samples on the basis of detection *p*-value [24] removal of cross-reactive CpGs [25], and the removal of CpGs containing polymorphic sites with a minor allele frequency > 1% at the target site or within the probe sequence. Background correction and dye-bias normalization was performed using the Noob method [26] contained in the *minfi* Bioconductor package. Between array-normalization was performed using the FunNorm procedure [27], which removes unwanted technical variation by regressing out variability explained by the control probes present on the array. In addition to background-correcting and normalizing the raw values, we performed BMIQ adjustment to correct for probe design bias [28] and subsequently used the ComBat procedure to adjust for batch effects. Altogether, the starting number of CpG loci was reduced from 485,577 to 428,936 loci.

When analyzing the association of serum dioxin level with methylation in the sperm tissue, dioxin level was modeled three ways: as a categorical variable (control vs. high), as a continuous variable using natural log-transformed dioxin, and as trend of dioxin (0 = control, 0.33 = low, 0.67 = medium, 1 = high). We used a linear model fit with an empirical Bayes method that adjusted for age, BMI, smoking, and dioxin levels, to determine the levels of methylation.

Data were initially analyzed using an epigenome wide association study (EWAS) approach to identify potential differentially methylated CpGs between control and high-dioxin-exposed subjects. To do this, surrogate variable analysis (SVA) was used to additionally remove any batch effects [29]. Employing the SVA package, we estimated the optimal number of surrogate variables was 5. Subsequently, linear models with empirical Bayes estimation adjusting for age, smoking, BMI, and the five surrogate variables were run. The Benjamini-Hochberg method was utilized to control the false discovery rate.

In order to select CpGs from the 450 K array that align with those from the Pilsner et al. study [17], which used reduced representation bisulfite sequencing, we employed the UCSC Genome Browser liftOver tool (https://genome.ucsc.edu/cgi-bin/hgLiftOver). Of the 666 loci found to be significantly associated with dioxin exposure, we were able to match 37 to our 450 K data. We repeated the SVA analysis on this subset of data, again using five surrogate variables.

Finally, we looked for effects within regions of DNA, rather than individual CpGs. DMRcate (version 1.18.0), which has previously been shown to perform favorably as compared competing methods for DNA methylation based regional-level analyses [30], was employed here to identify differentially methylated regions associated with serum dioxin levels. We used betas for all CpGs that were not within SNPs and ran DMRcate to calculate the average change for each region in beta values for control subjects compared to high exposure subjects, as well as both the Stouffer *p*-value [31] for regional-level statistical significance and the minimum adjusted p-value from the CpGs constituting the identified regions. We then sorted the 46,470 regions by the minimum adjusted p-value from the CpGs constituting the identified regions to identify putative regions with differing patterns of DNA methylation as a function of exposure level.

## Results

We obtained sperm methylation data for 37 members of the AFHS. Demographic data available from the participants included smoking status (ever vs. never), BMI, and age. We stratified the participants by serum dioxin levels (see Methods). Eleven of the veterans were classified as not exposed to Agent Orange and had a mean serum dioxin concentration of 4.3 pg/g lipid; four were exposed to low levels with a mean serum dioxin concentration of 6.5 pg/g lipid; eleven were exposed to medium levels with a mean serum dioxin concentration of 13.4 pg/g lipid; and eleven were exposed to high levels with a mean serum dioxin concentration of 61.1 pg/g lipid. The only significant difference between the four groups was the serum concentration of TCDD (Table 1).

**Table 1:** Demographics of the study population stratified by dioxin level

	Control (*N* = 11)	Low (*N* = 4)	Medium (*N* = 11)	High (*N* = 11)	All (*N* = 37)
Age [Mean (SD)]	70.7 (6.8)	79.5 (10.4)	77.0 (8.5)	68.9 (4.1)	73.0 (7.9)
Smoking: Never [N (%)]	2 (18.2)	1 (25.0)	3 (27.3)	4 (36.4)	10 (27.0)
Smoking: Former [N (%)]	8 (72.7)	3 (75.0)	7 (63.6)	5 (45.5)	23 (62.2)
Smoking: Current [N (%)]	1 (9.1)	0 (0.0)	1 (9.1)	2 (18.2)	4 (10.8)
BMI [Mean (SD)]]	32.4 (5.7)	25.7 (1.8)	30.9 (3.7)	29.6 (3.6)	30.4 (4.5)
Dioxin [Mean (SD), Range] (ppt)	4.3 (1.4), 2.2–7.1	6.5 (0.8), 5.6–7.2	13.4 (3.3), 8.9–19.6	61.1 (46.2), 26.6–167.6	24.1 (34.7), 2.2–167.6

**Fig. 1:**
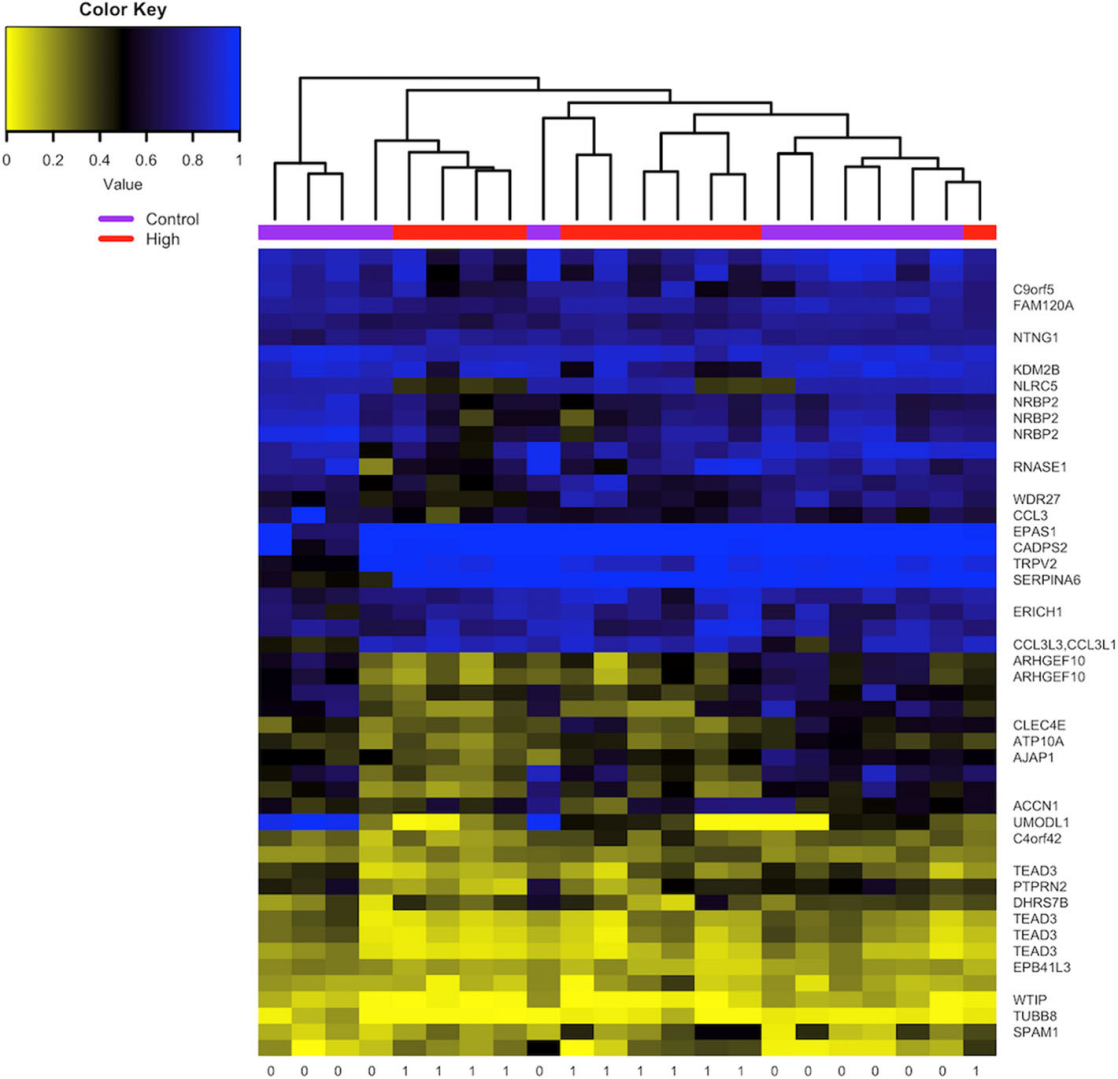
Heatmap of the unstructured cluster analysis of DNA methylation in sperm of high dioxin serum level Ranch Hand veterans compared with controls

Our initial agnostic approach to this data consisted of an epigenome-wide analysis, comparing the high exposed group with the low exposed. There were no CpGs with q < 0.05 after multiple testing adjustment, however several loci showed relatively large differences in methylation values between the highly exposed and the controls (Table 2). Of particular interest is the *TEAD3* gene where four different CpG sites showed similar loss of DNA methylation associated with dioxin exposure. The volcano plot resulting from a comparison of CpG-specific methylation values and dioxin exposure is shown in Additional file 1: Figure S1 while the heatmap of the unsupervised clustering analysis is shown in Fig. 1.

**Table 2:** Top 50 EWAS loci ranked by the change in beta value comparing highest exposed with control

Gene	CpG	CHR	Distance to TSS	Change in beta value	*P*-value	Mean beta in high expos	Mean beta in low expos
*UMODL1*	cg08880261	21	66	− 0.6	5.00E-04	0.24	0.53
*NLRC5*	cg03146649	16	175	−0.36	0.0013	0.6	0.8
*CCL3L3,CCL3L1*	cg03071134	17	2255	0.35	0.001	0.87	0.65
	cg16316180	10	3767	−0.31	4.00E-04	0.43	0.63
*SERPINA6*	cg11054397	14	2039	0.3	1.00E-04	0.98	0.8
	cg25345625	10	3889	−0.28	8.00E-04	0.34	0.51
	cg20710519	5	267	−0.26	0.0011	0.32	0.61
*NRBP2*	cg00061150	8	380	−0.26	0.0013	0.61	0.77
*TRPV2*	cg04702538	17	117	0.25	2.00E-04	0.93	0.85
*NRBP2*	cg24023418	8	534	− 0.24	6.00E-04	0.68	0.84
*KDM2B*	cg04682193	12	354	−0.24	0.0012	0.76	0.9
*SPAM1*	cg04471202	7	906	0.23	6.00E-04	0.31	0.17
*ERICH1*	cg02209998	8	384	0.22	4.00E-04	0.8	0.7
	cg04038019	20	501	−0.21	5.00E-04	0.71	0.84
*PTPRN2*	cg23887345	7	14,040	−0.21	8.00E-04	0.26	0.46
*C9orf5*	cg14364129	9	940	−0.2	9.00E-04	0.7	0.78
*TEAD3*	cg14909614	6	694	−0.2	0.0012	0.19	0.31
*NRBP2*	cg12515402	8	653	−0.19	4.00E-04	0.64	0.75
*ARHGEF10*	cg06807837	8	412	−0.19	1.00E-04	0.35	0.54
	cg14635160	9	3896	0.19	0.0014	0.25	0.12
	cg19356629	10	3037	−0.19	7.00E-04	0.74	0.86
*CCL3*	cg22518733	17	1973	−0.19	8.00E-04	0.57	0.66
*ARHGEF10*	cg07694735	8	348	−0.18	9.00E-04	0.32	0.5
	cg23154021	12	174	−0.18	0.0011	0.41	0.61
*TEAD3*	cg22424903	6	1059	−0.18	6.00E-04	0.11	0.22
	cg08576350	20	608	−0.17	4.00E-04	0.76	0.89
*TEAD3*	cg01487910	6	563	−0.17	6.00E-04	0.14	0.24
	cg14181345	9	1634	−0.17	3.46E-05	0.65	0.72
*CADPS2*	cg23390958	7	315	0.16	3.00E-04	0.99	0.92
*AJAP1*	cg21159274	1	808	−0.15	0.0011	0.42	0.52
*ACCN1*	cg15744026	17	1045	0.14	0.001	0.53	0.51
*TEAD3*	cg01546820	6	263	−0.13	5.00E-04	0.08	0.16
*WDR27*	cg00392003	6	2873	−0.12	4.00E-04	0.6	0.68
*EPAS1*	cg09119656	2	533	0.12	2.00E-04	0.99	0.94
	cg27511894	1	544	0.12	0.0014	0.82	0.76
*CLEC4E*	cg12762799	12	410	−0.12	6.00E-04	0.39	0.45
	cg05240760	2	75,792	0.11	7.00E-04	0.78	0.78
*ATP10A*	cg07986058	15	26	−0.11	0.0011	0.34	0.41
*EPB41L3*	cg01673082	18	2874	−0.1	4.70E-05	0.14	0.21
	cg06654337	6	12,717	−0.6	5.00E-04	0.15	0.16
*WTIP*	cg05620261	19	804	−0.36	0.001	0.02	0.12
*FAM120A*	cg15886017	9	322	0.35	5.77E-05	0.77	0.83
*DHRS7B*	cg06582708	17	945	−0.31	4.00E-04	0.33	0.31
*C4orf42*	cg21793579	4	1100	0.3	1.00E-04	0.28	0.28
*RNASE1*	cg05958352	14	789	−0.28	8.00E-04	0.69	0.76
*NTNG1*	cg14787581	1	1367	−0.26	0.0011	0.79	0.76
	cg12951997	12	1369	−0.26	0.0013	0.88	0.91
*TUBB8*	cg09677976	10	1317	0.25	2.00E-04	0.03	0.05
	cg07320695	4	5635	−0.24	6.00E-04	0.26	0.22
	cg19648967	14	2607	−0.24	0.0012	0.72	0.76

Linear models were run with empirical Bayes estimates adjusting for age, smoking, BMI, and five surrogate variables, with log-transformed dioxin as the predictor. All 37 subjects were included in this analysis. We found associations of methylation with TCDD levels in 24,548 of 428,936 loci (5.7%), but none were statistically significant at the 5% FDR level. The majority of loci were positively associated with dioxin; that is, higher levels of dioxin were associated with higher levels of methylation. Effect levels ranged from − 0.23 to 0.17 change in methylation beta-values per unit increase in log dioxin.

Since our power to detect epigenome-wide differences in DNA methylation associated with Agent Orange exposure was quite low, a candidate-gene approach was also conducted, comparing our data with that of Pilsner et al [17], who studied sperm of dioxin-exposed Russian boys. The Ranch Hand methylation data was directly compared to that of Russian Children’s Study after extracting overlapping loci. Since the Russian Children were studied using Reduced Representation Bisulfite Sequencing (RRBS) only 37 of the 666 CpGs reported to be associated with dioxin exposure in that report were available for analysis. After completing a t-test for the Ranch Hand data at each of the 37 overlapping loci, we found five to have a significant difference in methylation (*p* < 0.05) between control and high exposure groups, and two additional loci to be marginally significant before correction for multiple comparisons (*p* < 0.1; Table 3). After FDR correction none of the loci were statistically significant (Table 3). Several genes contained more than one of the 37 overlapping CpGs, with *SAMD11* housing the most (*N* = 4) followed by *PGBD5* (*N* = 3). Other genes that contained multiple overlapping CpGs with the Russian Children’s Study include *KCNN1*, *FAM83C*, and *RBM33*. To test for the directionality of the methylation at each of these sites, we compared the difference in methylation beta values between the high and low exposure groups (Table 3). Interestingly, the 3 sites associated with the *PGBD5* gene, when compared by dioxin exposure level in the Ranch Hand participants with the data from the Russian boys [17], were all statistically significantly altered before FDR correction and showed common directionality. The *MBP* locus showed evidence of significant demethylation and the two sites in the *KCNN1* locus were borderline significantly altered, and in each case the change was in the same direction as reported in Pilsner et al [17]. The remaining sites were either not significantly altered or showed inconsistent directional change when compared with the Pilsner et al [17] data. We also examined the AhR pathway, since dioxin is known to interact with genes in this pathway, for alterations in methylation of these loci that correlated with TCDD level but did not find any of statistical significance (data not shown).

**Table 3:** Mean DNA methylation in sperm by exposure group in 37 candidate CpG loci

Gene	CpG	Russian Low Exposure Group	Russian High Exposure Group	Ranch Hand Low Exposure	Ranch Hand High Exposure	*P*-value	FDR adjusted *p*-value
*SAMD11*	cg06624358	0.58	0.92	0.66	0.61	0.72	0.81
*SAMD11*	cg14156792	0.51	0.97	0.60	0.55	0.77	0.81
*SAMD11*	cg01727431	0.59	1	0.59	0.53	0.67	0.80
*SAMD11*	cg02439789	0.46	0.83	0.58	0.49	0.51	0.80
*PGBD5*	cg11570508	0.92	0.72	0.97	0.92	0.047	0.35
*PGBD5*	cg11226042	0.97	0.58	0.93	0.87	0.039	0.35
*PGBD5*	cg22875527	0.95	0.61	0.95	0.89	0.027	0.5
*CCNY*	cg05701548	0.14	0.38	0.57	0.55	0.77	0.81
*RBP3*	cg07070043	0.76	0.34	0.66	0.56	0.035	0.35
	cg26066724	0.88	0.32	0.75	0.65	0.34	0.63
	cg07458849	0.91	0.80	0.88	0.87	0.87	0.87
*SLC5A10*	cg04585390	0.60	0.35	0.58	0.52	0.34	0.63
*MBP*	cg00706570	0.76	0.40	0.72	0.47	0.005	0.185
	cg06167221	0.69	0.80	0.79	0.69	0.12	0.40
	cg00593773	0.61	0.82	0.85	0.77	0.10	0.40
*KCNN1*	cg21491340	0.17	0.07	0.38	0.30	0.096	0.40
*KCNN1*	cg21491340	0.14	0.05	0.38	0.30	0.096	0.40
*FBLN7*	cg11229230	0.83	0.96	0.98	0.97	0.20	0.60
*DGKD*	cg24933152	1	0.92	0.9778	0.9818	0.060	0.37
*FAM83C*	cg12170299	0.78	0.72	0.67	0.63	0.27	0.63
*FAM83C*	cg11133013	0.858	0.63	0.73	0.64	0.21	0.60
*C21orf29*	cg12061357	0.61	0.25	0.60	0.51	0.12	0.40
	cg26311420	0.56	0.15	0.40	0.45	0.66	0.80
	cg26311420	0.5	0.13	0.40	0.45	0.66	0.80
*RBM33*	cg14353208	0.82	0.99	0.98	0.97	0.48	0.80
*RBM33*	cg07262012	0.80	0.97	0.96	0.96	0.53	0.80
	cg14720951	0.28	0.14	0.13	0.17	0.34	0.63
	cg06182811	0.75	0.73	0.69	0.74	0.31	0.63
	cg06182811	0.85	0.74	0.69	0.74	0.31	0.63
	cg19673480	0.83	0.64	0.77	0.79	0.64	0.80
	cg19673480	0.82	0.59	0.77	0.79	0.64	0.80
	cg23541013	0.71	0.44	0.72	0.77	0.33	0.63
	cg01695620	0.74	0.61	0.83	0.87	0.43	0.76
	cg15690180	0.55	0.19	0.46	0.49	0.77	0.81
*FLJ43860*	cg12732638	0.65	0.84	0.84	0.85	0.79	0.81
*PLEC1*	cg04247530	0.86	0.94	0.84	0.82	0.55	0.80
*TBC1D2*	cg14049807	0.06	0.26	0.43	0.39	0.62	0.80

**Fig. 2:**
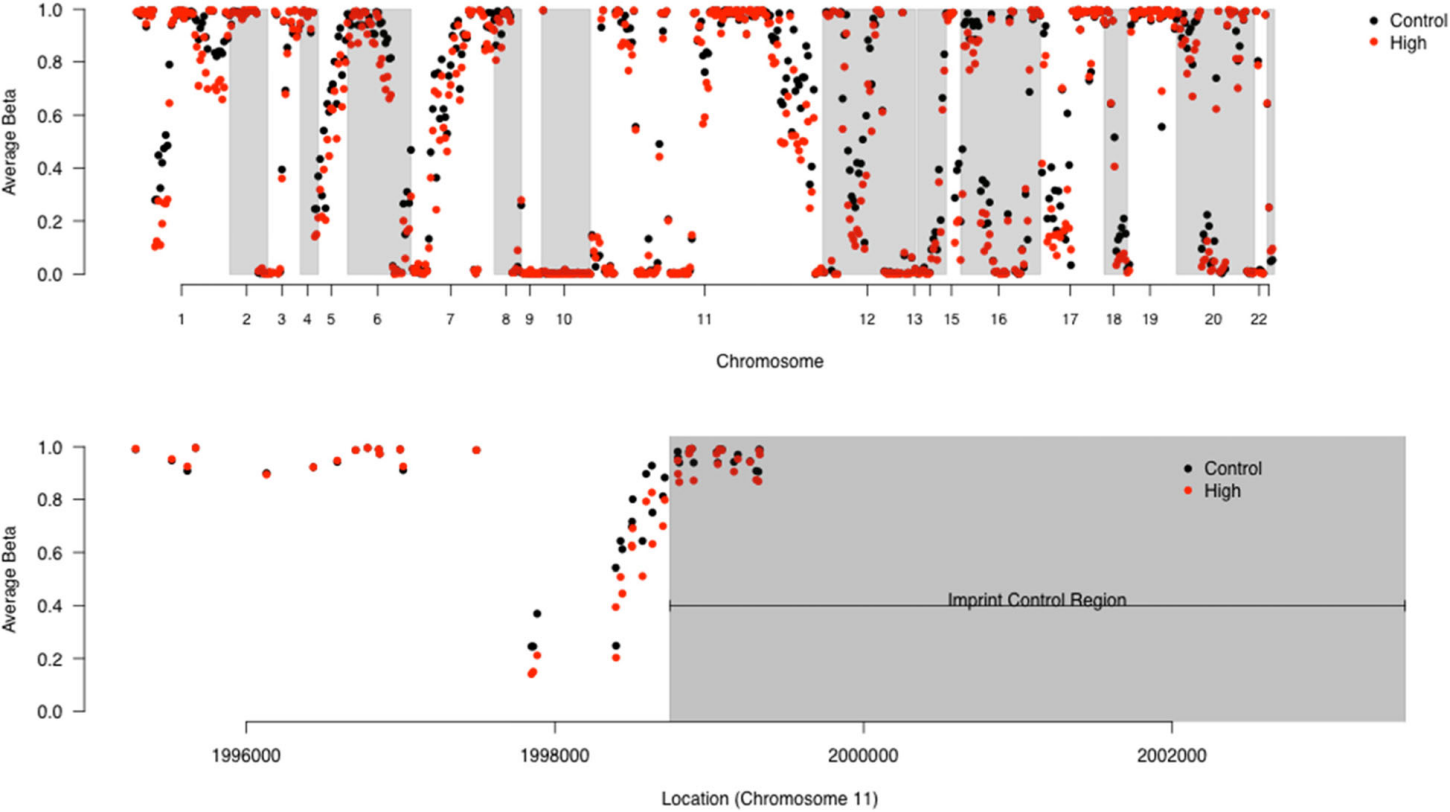
Manhatten plot of the regions where alterations in DNA methylation were significantly associated with dioxin serum level

Due to the low power to find significant associations in CpG by CpG analyses, DMRcate software was also employed to similarly examine coordinate alterations in gene regions that associate with TCDD level. While none of the of the 46,470 regions examined resulted a statistically significant Stouffer *p*-value after adjustment for multiple testing, we did observe 31 regions with minimum adjusted p-value < 10^− 6^ from the CpGs constituting the region. Of these regions, 5 were located in chromosome 11, including a region with a p-value of < 10^− 28^ which is associated with the imprinted gene H19 and contains 50 CpGs. Most of the regions (26 out of 31) showed hypomethylation for high dioxin subjects compared to controls (Fig. 2), including the most significant region for which the average beta value was 0.15 lower (on the beta-value scale) for highly exposed subjects compared to controls. Interestingly, the variation in DNA methylation was most evident at the site of the imprinting control region (Fig. 2). The complete list of regions where altered patterns of DNA methylation were significantly associated with AO exposure are shown in Table 4.

**Table 4:** CpG regions (by P-Value) identified as altered in DMRcate analysis of Agent Orange exposed compared with control

CHR	Start	End	Width	# CpGs	*P*-value	Stouffer Adjusted *p*-value	Max beta-fold change	Mean beta-fold change	Overlapping Genes
11	2,016,513	2,020,560	4048	50	1.83E-29	1	−0.13	− 0.04	*H19*
19	35,530,254	35,532,097	1844	9	5.77E-15	0.93	−0.21	−0.15	*HPN*
1	32,041,052	32,042,921	1870	12	8.02E-15	0.99	− 0.15	− 0.08	*TINAGL1*
20	62,205,530	62,205,981	452	9	6.21E-14	0.93	−0.12	− 0.09	*HELZ2*
20	57,581,478	57,583,709	2232	27	3.21E-13	1	−0.05	− 0.01	*CTSZ*
17	75,315,081	75,317,185	2105	15	2.27E-12	0.99	−0.15	−0.07	*SEPT9*
11	2,441,045	2,442,304	1260	7	4.08E-12	0.91	−0.16	−0.14	*TRPM5*
22	37,640,250	37,641,506	1257	7	5.82E-12	0.92	−0.11	−0.09	*RAC2*
19	6,772,370	6,773,074	705	4	2.23E-11	0.84	−0.12	−0.08	*VAV1*
5	2,007,375	2,008,713	1339	8	1.16E-10	0.92	−0.15	−0.13	*NA*
16	725,416	730,187	4772	17	1.66E-10	1	−0.07	−0.01	*STUB1, RHBDL1, LA16c*
16	4,729,905	4,733,181	3277	14	1.73E-10	1	−0.2	−0.05	*MGRN1*
18	74,691,097	74,692,265	1169	9	3.00E-10	0.93	−0.2	−0.17	*RP11*
1	6,129,242	6,134,427	5186	10	5.30E-10	0.99	0.03	0	*NA*
14	74,823,368	74,824,804	1437	8	7.83E-10	0.92	−0.11	−0.07	*NA*
20	60,540,388	60,541,082	695	5	9.37E-10	0.87	−0.17	−0.15	*NA*
6	169,653,612	169,655,497	1886	12	1.24E-09	0.98	−0.13	−0.06	*THBS2*
1	6,085,799	6,086,652	854	9	1.59E-09	0.95	−0.2	−0.16	*KCNAB2*
2	495,912	497,296	1385	7	9.03E-09	0.96	−0.17	−0.1	*NA*
12	133,463,607	133,465,592	1986	31	1.96E-08	1	0.08	0.01	*CHFR, RP11*
11	2,554,198	2,555,576	1379	11	2.30E-08	0.98	−0.14	−0.05	*NA*
9	124,497,763	124,499,870	2108	6	2.73E-08	0.94	−0.02	−0.01	*NA*
17	68,171,276	68,171,750	475	3	5.25E-08	0.78	0.01	0.01	*NA*
5	6,775,909	6,775,922	14	2	2.52E-07	0.76	−0.21	−0.2	*NA*
1	203,840,145	203,840,808	664	2	2.71E-07	0.76	0.02	0.01	*NA*
7	100,727,888	100,729,412	1525	10	5.27E-07	0.96	−0.1	−0.07	*TRIM56*
11	68,778,570	68,782,202	3633	19	5.73E-07	1	−0.08	−0.03	*MRGPRF, RP11*
1	203,320,190	203,320,541	352	5	6.54E-07	0.87	−0.15	−0.12	*FMOD*
7	73,441,057	73,443,113	2057	8	7.83E-07	0.95	−0.12	−0.08	*ELN*
11	3,186,095	3,188,016	1922	28	8.03E-07	1	−0.09	−0.04	*OSBPL5*
17	790,628	791,324	697	5	9.74E-07	0.99	0.02	0.01	*NA*

## Discussion

We have directly investigated the association of exposure to the dioxin-containing herbicide toxicant Agent Orange, widely used during the Vietnam War, with demonstrable alterations in the epigenome of sperm. AO was highly contaminated with TCDD, which is known to be an endocrine disrupting chemical, and evidence is accumulating that it is associated with abnormal reproductive health endpoints. Our study of a small number of AO-exposed Operation Ranch Hand veterans enrolled in the Air Force Health Study found no evidence of any statistically significant exposure-associated alterations in DNA methylation when studied genome-wide in approximately 450,000 loci. However, there were genes, such as *TEAD3*, with multiple loci seemingly coordinately altered in the DNA from sperm in the exposed compared with control veterans. When we examined methylation at genes previously reported to be altered by dioxin exposure in prepubertal boys, we observed several loci with significant differences in methylation comparing the control and high exposed groups; 3 loci in the *PGBD5* gene were significantly altered in the same direction as the prior study and loci in the *MBP* and *KCNN1* gene loci were similarly altered in a fashion comparable to that previously reported by Pilsner et al [17]. However, none of the above findings survived correction for false discovery and thus were of limited statistical significance. At the same time, most remarkably, when we analyzed regional differences in methylation, there were numerous CpG stretches containing CpGs that were coordinately differentially altered after exposure, including one dramatic change in the imprinted *H19* region on chromosome 11 (minimum adjusted *p* = 10^− 28^).

*H19* is a noncoding imprinted gene that is transcribed to form a microRNA (miR-675) [32] thought to influence early development. Neighboring *H19* on the chromosome is another imprinted gene, *IGF2*, which is also implicated in embryogenesis. These genes are regulated by an imprinting control region that dictates the expression of *H19* exclusively on the maternal allele, and *IGF2* on the paternal allele. On the maternal side *IGF2* is normally unmethylated, allowing the binding of the zinc-finger CTCF protein which blocks its expression [6, 32]. On the other hand, *H19* on the paternal side tends to be hypermethylated which enables the expression of *IGF2* [33]. Interestingly, the *H19* locus has previously been shown to be sensitive to alteration by TCDD; exposure to mouse preimplantation embryos causes hypermethylation and decreased expression of the *H19* gene [6]. However, male mice born from a mother exposed to TCDD showed no difference in methylation in imprinted genes, but increased expression of those genes [34]. Other studies, in rodents, of reproductive toxicity have shown TCDD exposure of pregnant animals to be associated with H19-mediated cleft palate [35, 36], as well as with H19-mediated ovarian toxicity [37]. Studies of epigenetic alterations to the *H19* gene in men have been limited; however, an investigation into the sperm epigenome of males who recurrently fathered miscarriages found the *H19* ICR and CTCF6 protein binding region to be hypomethylated [32]. Hypomethylation in these loci could cause the binding of the CTCF protein on the paternal allele rather than the maternal allele, thus blocking the expression of *IGF2* [32]. Similarly, the *H19* gene in sperm has been found to be hypomethylated in a cohort of males who practice chronic alcohol consumption [38]. Hence, while this locus seems to be clearly sensitive to DNA methylation alteration by TCDD exposure, the nature of the change (e.g. hyper vs hypomethylation, for example) may be related to the lifestage at exposure (e.g. in-utero compared with adolescent or adult exposure), to the kind of exposure (acute vs chronic) or to other parameters that remain unexplored and unknown.

Little data is available concerning the remaining gene regions found to be associated with serum dioxin level in the regional analysis. Further study of these regions would seem warranted. However, the *TEAD3* gene, which we observed to be associated with TCDD level in the EWAS analysis, is a transcription factor known to interact with the *YAP* gene product, to participate in regulating the HIPPO pathway [39]. HIPPO signaling has been shown to be important in development of mature sperm in sheep, but its role in human sperm remains to be elucidated [40]. Hence, the significance of any association of alterations in this gene and TCDD exposure is unclear. The *PGBD5* gene codes for a transposase enzyme [41] and it is possible that silencing this gene is important in maintaining the genomic stability of germ cells that undergo demethylation during development. Exposure in both studies was associated with loss of methylation at these loci. However, this finding clearly requires further investigation to fully understand its significance.

The impact of dioxin exposure on the reproductive health has been widely studied in animal models as well as exposed humans. In utero exposure to TCDD is known to decrease epididymal sperm count, fertility and testosterone levels in rats [42]. Similar exposure of mice also has been reported to induce transgenerational epigenetic alterations appreciable as changes in DNA methylation in sperm [43]. Interestingly, TCDD-exposed adult rats showed marked histological abnormalities in the testes, changes in estrogen and testosterone levels, and an altered testicular proteome [44]. Another study of adult rats exposed to TCDD showed decreased serum testosterone levels, fewer Sertoli cells, and reduced sperm production [45].

Studies in humans have produced conflicting results. Twenty-two years after a factory explosion in Seveso, Italy that released large amounts of TCDD, men who were exposed at prepubertal ages showed decreased sperm concentrations and motility [16]. Similarly, a prospective study of a group of Russian boys aged 8–9 exposed to dioxins through contamination in their community found that 10 years after first exposure, higher serum TCDD concentrations were associated with decreased sperm concentrations, counts, and motility [15]. Especially in terms of sperm parameters, dioxins seem to negatively impact the reproductive health of men who were exposed at young ages. Taken together, it is thought that during the developmental stages of life, individuals are several orders of magnitude more susceptible to the effects of TCDD, resulting in pronounced consequences to fertility even at low exposures [46].

The effects of dioxin exposure on reproductive health in men exposed in puberty and adulthood is less clear. TCDD appeared to serve as a stimulant to semen production in the men from Seveso, Italy, who were exposed during puberty, and those exposed as adults showed no differences from the control group [16]. A study of men living in areas of Vietnam with high dioxin contamination found no difference in prostate-specific antigen concentrations between control and high serum dioxin groups [47]. Also, the Vietnamese men with high serum dioxin showed increased testosterone and estradiol levels, but decreased dehydroepiandrosterone levels, and no change in other steroid hormones [48]. Exposure to dioxin-contaminated food was associated with a decrease in total and free testosterone [49], although (as noted above) several studies have not found TCDD exposure to alter semen quality or sperm count (reviewed in [50]). Similarly, Hsu and colleagues [51] prospectively studied adult Taiwanese men exposed to dioxins through food, finding lower sperm concentrations, increased incidence of abnormal sperm morphology, and reduced ability of spermatozoa to penetrate hamster oocytes. We studied veterans who were young adults at the time of exposure (in their 20s) and thus may have been at the limits of or beyond any putative ‘window of susceptibility’ to the induction of epigenetic changes by dioxin. As noted above, the veterans that were studied here did not demonstrate any changes in semen analysis, specifically including sperm count and quality.

This work has several limitations. Perhaps most importantly, we were quite underpowered to find epigenome-wide alterations in sperm associated with AO exposure, since we studied only 37 veterans. Additionally, while TCDD exposure occurred from 1962 to 1971, participants were not initially studied until 1982 [19]. Given that the half-life of TCDD in humans is 7 to 11 years [1], the measured serum dioxin levels were lower than those incurred during the exposure period. This, along with measurement error associated with the device/instrument used to measure serum dioxin levels, also could have affected our ability to detect induced alterations. While we attempted to adjust for all compounding factors, the latency of our analysis following the initial exposure allowed for a large window of time for uncontrolled factors to make an impact. Although we used a genome-wide platform for assessing DNA methylation, it was limited in its interrogation of loci studied using different methods (e.g. Pilsner et al [17] who used RRBS).

In summary, studying a small number of sperm samples from AO-exposed Ranch Hand veterans enrolled in the AFHS, we did not find evidence of significant epigenome-wide alterations associated with AO exposure. However, we did replicate several of the findings of a prior study of dioxin-exposed Russian boys in an analysis restricted to study of overlapping loci on the 2 platforms. Additionally, we produced evidence that the *H19* gene region, among others, is altered in the sperm of Ranch Hand veterans. Due to the importance of this regions association with adverse reproductive outcomes and the suggestion of other identified regions to be similarly impacted by Agent Orange exposure, further investigation is warranted.

## Supplementary information

**Additional file 1: Figure S1.** Volcano plot of the comparison of CpG-specific methylation values and dioxin exposure.

## Data Availability

The data are available on GEO (pending acceptance).

## Abbreviation

AFHS: Air Force Health Study
