# Supplementary material for: Serum dioxin and DNA methylation in the sperm of operation ranch hand veterans exposed to Agent Orange
Source: Environ Health. 2019 Oct 29;18:91. doi: 10.1186/s12940-019-0533-z (PMC6819394; doi:10.1186/s12940-019-0533-z)

**Additional file 1: Figure S1 – Volcano plot of the comparison of CpG-specific methylation values and dioxin exposure**


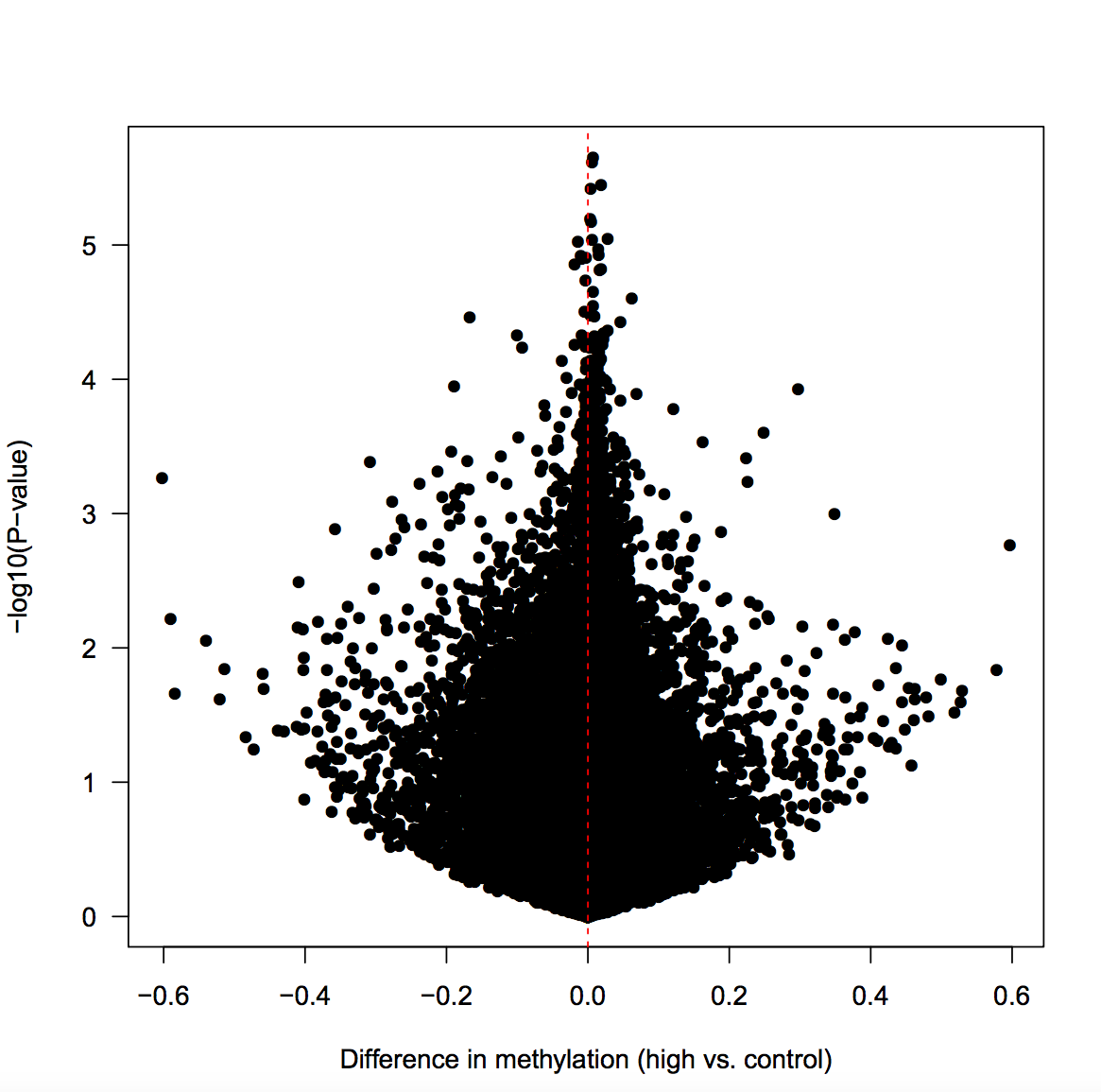

Supplement: Supplementary file 1 — Additional file 1: Figure S1. Volcano plot of the comparison of CpG-specific methylation values and dioxin exposure. [file 12940_2019_533_MOESM1_ESM.docx]
